# The effects of omega-3 fatty acids on Wnt activity in iPSC-derived neural stem cells from adult ADHD patients

**DOI:** 10.1016/j.nsa.2026.107005

**Published:** 2026-05-02

**Authors:** Cristine M. Yde Ohki, Natalie M. Walter, Louisa C. Dury, Letizia Del Campana, Romane Lasmarrigues, Rhiannon V. McNeill, Zora Schickardt, Lukasz Smigielski, Susanne Walitza, Sarah Kittel-Schneider, Edna Grünblatt

**Affiliations:** a Department of Child and Adolescent Psychiatry and Psychotherapy, Translational Molecular Psychiatry, University Hospital of Psychiatry Zurich, University of Zurich, Wagistrasse 12, 8952, Schlieren, Switzerland; b Department of Psychiatry, Psychosomatics and Psychotherapy, Center of Mental Health, University of Würzburg, Würzburg, Germany; c Neuroscience Center Zurich, University of Zurich and the ETH Zurich, Winterthurerstrasse 11, 8057, Zurich, Switzerland; d Zurich Center for Integrative Human Physiology, University of Zurich, Winterthurerstrasse 11, 8057, Zurich, Switzerland; e Department of Psychiatry and Neurobehavioural Science, University College Cork, Cork, Ireland; f APC Microbiome, University College Cork, Cork, Ireland

**Keywords:** Attention-deficit/hyperactivity disorder, Neural stem cells, Wnt signaling, Omega-3, Docosahexaenoic acid, Methylphenidate

## Abstract

The canonical Wnt signaling pathway plays a key role in neurodevelopment and in maintaining cellular homeostasis of the central nervous system in adulthood. It regulates essential processes, such as cell proliferation, differentiation, and synaptogenesis. *In vivo* and genetic evidence suggests an association between attention-deficit/hyperactivity disorder (ADHD) and abnormalities in the Wnt pathway. Prior research from our group indicated heightened basal Wnt activity in neural stem cells (NSCs) derived from induced pluripotent stem cells (iPSCs) of pediatric ADHD patients and showed that methylphenidate (MPH) and omega-3 polyunsaturated fatty acids (ω-3 PUFAs) can modulate this pathway. While MPH is a first-line treatment for ADHD, ω-3 PUFAs are under investigation as a potential non-pharmacological supplement. Using the same functionality-based reporter technology, we assessed Wnt activity in iPSC-derived NSCs from 5 adult ADHD patients and 5 healthy controls. We examined whether ω-3 fatty acids (EPA and DHA), alone or combined, and DHA with MPH can modulate Wnt activity in these NSCs. Basal Wnt responsiveness did not differ between adult ADHD and control lines, although genetic risk for ADHD was associated with increased Wnt activity. EPA showed no statistically significant effects, while DHA strongly increased Wnt activity in both groups. DHA combined with EPA increased Wnt activity in ADHD lines, whereas DHA with MPH caused group-specific responses: enhancing Wnt signaling in ADHD but reducing it in controls. Compared to NSCs derived from children, adult-derived NSCs behave differently and show distinct responses to EPA and DHA, suggesting a potential route toward refined therapeutic strategies for adults.

## 1 Introduction

Despite its neurodevelopmental nature, attention-deficit/hyperactivity disorder (ADHD) persists into adult life in around 60% of cases worldwide (Sibley et al., 2017). ADHD's multifactorial etiology, characterized by a complex interaction of genetic and environmental factors, remains unresolved, with heritability rates ranging from 77% to 88% (Faraone and Larsson, 2019).

The psychostimulant methylphenidate (MPH) is widely used as the first recommendation in many guidelines for treating ADHD (Diagnosis and management guideline (NG87), 2019; AWMF, 2017). Its best-known mechanism of action involves blocking dopamine and norepinephrine transporters in presynaptic neurons (Kuczenski and Segal, 1997). Clinically, MPH leads to symptomatic improvements and has been linked to amelioration of structural and functional brain maturation delays observed in some patients (Castellanos et al., 2002; Frodl and Skokauskas, 2012; Hart et al., 2013; Nakao et al., 2011; Sobel et al., 2010; Shaw et al., 2009). However, approximately 30% of patients exhibit no or only partial response to MPH (Gajria et al., 2014; Quintero et al., 2010), and in adulthood, cardiovascular risks may restrict its use (Eroglu et al., 2023; Garcia-Argibay et al., 2024). It is therefore essential to explore novel therapeutic strategies, including non-pharmacological approaches such as omega-3 polyunsaturated fatty acids (ω-3 PUFAs).

Interest in ω-3 as a potential anti-inflammatory therapeutic option for ADHD has increased in light of several promising findings. For instance, ω-3 deficiency has been suggested as a potential risk factor for ADHD (Richardson, 2006), and its supplementation has been shown to improve ADHD symptomatology in patients (Chang et al., 2018; Sonuga-Barke et al., 2013). Furthermore, the combination of MPH and ω-3 has been reported to show tolerability comparable to monotherapy with MPH, whilst also allowing a reduction in the MPH dose (Barragán et al., 2017). However, more research into this field is required. Both MPH and ω-3 PUFAs have been described as modulators of the canonical Wnt signaling pathway in neural cells (ShamsEldeen et al., 2021; Zhao et al., 2019; Grünblatt et al., 2018). Alongside other genetic and *in vivo* evidence, these findings suggest that the canonical Wnt signaling pathway is implicated in ADHD, as reviewed by our group in 2020 (Yde Ohki et al., 2020). Moreover, we recently reported disruptions in Wnt signaling in neural cell lines from children and adolescents diagnosed with ADHD, as well as modulatory effects of MPH and ω-3 (Walter et al., 2024, 2025).

In induced pluripotent stem cell (iPSC)-derived neural stem cells (NSCs) from ADHD patients, our group previously demonstrated that Wnt activity is higher in ADHD NSCs derived from children and adolescents aged 6‒18 who respond clinically to MPH treatment (Walter et al., 2024). Furthermore, these NSCs exhibited impaired cell proliferation, a Wnt-modulated cellular process (Yde Ohki et al., 2023a, 2025). Earlier research from our group demonstrated that treatment with MPH at 10 nM and eicosapentaenoic acid (EPA) at 50 μM treatments can alter Wnt activity in NSCs from pediatric ADHD patients who show clinical response to MPH. In addition, the combination of docosahexaenoic acid (DHA) and MPH enhanced Wnt activity in both MPH responder and healthy control cell lines, while a 3:1 EPA:DHA ratio increased Wnt activity in NSCs from MPH non-responders (Walter et al., 2025). Taken together, these findings show that Wnt activity can be modulated in a group-specific manner.

Alterations in this cellular pathway have also been described in other neurodevelopmental disorders (e.g. autism spectrum disorders and Pitt-Hopkins Syndrome), as demonstrated using iPSC-derived models (Papes et al., 2022; Marchetto et al., 2017; Sawada et al., 2020). These studies hypothesized that Wnt abnormalities may affect cellular phenotypes such as cell proliferation and ultimately have clinical implications. Concerning ADHD, Demontis et al. have recently reported Wnt-related rare genetic variants (i.e., in the *LRP1* and *WNT1* genes) associated with a higher risk of develop ADHD after analyzing the whole-exome genome-sequencing data from 8895 ADHD patients and 9001 controls (Demontis et al., 2025).

To investigate whether adults with persistent ADHD exhibit alterations in Wnt activity comparable to those observed in pediatric ADHD patients, and consequently may benefit from PUFA treatment, we derived NSCs from adult ADHD patients and matched healthy controls. We examined whether treatments with MPH and ω-3 PUFA, specifically DHA and EPA, could modulate Wnt activity. In this context, a comprehensive understanding of the underlying molecular and cellular mechanisms, including Wnt signaling, may be crucial for elucidating ADHD pathophysiology and identifying novel therapeutic strategies.

## 2 Methods

### 2.1 Subject recruitment

Adult ADHD patients (aADHD) and healthy controls (aControl) were recruited at the Department of Psychiatry, Psychosomatics and Psychotherapy, University Hospital Würzburg (Germany), and the Department of Psychiatry, Psychosomatic Medicine and Psychotherapy, University Hospital Frankfurt (Germany).

As described in McNeill et al. (2026), ADHD diagnoses were established independently by two psychiatrists according to the Diagnostic and Statistical Manual of Mental Disorders, Fourth Edition (DSM-IV), or Fifth Edition (DSM-5) criteria, supported by and self-report surveys tailored to ADHD, the Wender Utah Rating Scale – short form (WURS-k) (Retz-Junginger et al., 2002) and the ADHD Self-Report Scale (ADHS-SB) (Kessler et al., 2005). Healthy controls without any severe neurological, internal, infectious, or mental conditions were recruited and mental disorders were ruled out using the Mini-DIPS screening questionnaire (Margraf and Cwik, 2017). The study was approved by the Ethical Committees of the University of Würzburg (#96/11) and the University of Frankfurt (#425/14). All participants have provided signed informed consent forms.

In this study, cell lines from 5 ADHD patients and 5 neurotypical controls (aged 25‒53 years) were investigated. Additional demographic information about these individuals are provided in Supplementary Table 1.

### 2.2 Calculation of ADHD Polygenic Risk Scores

To determine genetic liability for ADHD, individual Polygenic Risk Scores (PRS) were computed using a clumping/thresholding approach (*p* = 0.05) in PLINK. Genotyping was performed on DNA derived from fibroblasts of each individual, and PRS were computed based on the summary statistics from the latest ADHD-related Genome Wide Association Studies (GWAS) by Demontis et al., 2023. All samples were genotyped at Life and Brain GmbH (University of Bonn) using the Infinium Global Screening Array (Illumina).

### 2.3 Generation of iPSC-derived NSCs

Fibroblast-derived iPSCs were generated using Sendai virus transduction (CytoTune-IPs 2.0 Sendai Reprogramming Kit, Invitrogen), quality controlled (QC) and cultured on Matrigel (Corning)-coated plates until the experimental setup, as previously described (McNeill et al., 2023; McNeill et al., 2026; Palladino et al., 2018). These cells underwent QC steps reported previously (McNeill et al., 2023;McNeill et al., 2026; Palladino et al., 2020). Unpublished characterization of several iPSCs investigated in this study (CTL1, CTL3, ADHD1, ADHD3 and ADHD4) is shown in Supplementary Fig. 1. For further details, see Supplementary Table 1.

All iPSCs were subjected to a 7-day neural induction protocol using PSC Neural Induction Medium (Gibco) in Matrigel-coated wells to generate NSCs from each line (Yde Ohki et al., 2023c). The NSCs were maintained in Neural Expansion Media (NEM) until passages 3‒5 for QC experiments, including RT-qPCR and immunocytochemistry (Supplementary Fig. 2).

For RT-qPCR, *ACTB* and *REEP5* were utilized as reference genes to assess the gene expression of NSC markers: *SOX2*, *Nestin* and *PAX6*. For immunocytochemistry, the positive expression of FOXG1, PAX6, TUBB3, SOX2, and Nestin was qualitatively verified similar to previous reports (Walter et al., 2024; Yde Ohki et al., 2023a, 2023b, 2025).

### 2.4 Wnt-reporter assay

Our group has developed a cell-based Wnt-reporter assay, based on the detection of Firefly luciferase (*luc2P*), a reporter gene under the control of a TCF/LEF responsive element, as previously described (Yde Ohki et al., 2023b). In the canonical Wnt/β-catenin pathway, transcriptional activation by TCF/LEF is the central readout of signaling activity, providing the biological basis for this assay. Briefly, activation of the Wnt signaling pathway is triggered by the binding of extracellular Wnt ligands (e.g., Wnt3a, Wnt7a) and/or the absence of antagonists (such as DKK1) to the transmembrane receptors LRP5/6 and Frizzled. This process leads to inhibition of the multiprotein destruction complex, which is no longer able to phosphorylate β-catenin for subsequent degradation by proteasomes (Aberle et al., 1997). The effector β-catenin accumulates in the cytoplasm and can translocate into the nucleus, where it activates transcription of target genes after binding to the TCF/LEF family of transcription factors (MacDonald et al., 2009). Our reporter system exploits this final transcriptional step: TCF/LEF-mediated activation directly induces Firefly luciferase expression, providing a quantitative readout of Wnt pathway activity.

Briefly, 20,000 NSCs were seeded in Matrigel-coated 96-wells, in triplicate. On the following day, the cells were co-transfected with a plasmid containing the Firefly luciferase gene and a normalization vector, carrying the NanoLuc® luciferase gene under the control of a constitutive Thymidine Kinase (TK) promoter.

After 24 h, the transfected cells were treated with Wnt3a or DKK1. Increasing concentrations of the Wnt agonist Wnt3a (0, 5, 10, 20, 40, 60, 80, 100, 150, 200, 400, 600, and 800 ng/mL) were added to the NSCs cultures. The EC_50_ for each cell line was subsequently calculated. For the antagonist DKK1, each cell line was first individually treated with its respective EC_25_ concentration of Wnt3a and incubated for 10 min at 37 °C and 5% CO_2_. DKK1 (0, 4, 10, 20, 30, 40, and 50 ng/mL) was then added to the cultures. After an overnight incubation at 37 °C and 5% CO_2_, luminescence assays were performed and dose-response curves were plotted to determine the IC_50_ values for DKK1. This procedure is described in detail in 40.

Next, we tested the effects of the compounds of interest. 10 nM MPH, 25 μM and 50 μM DHA and EPA were administered individually, while the combinations of 10 nM MPH +25 μM DHA and 8.3 μM DHA +16.7 μM EPA (1:3) were also assessed, according to the methodology described in Walter et al. (2025). For the combined DHA + EPA condition, the two fatty acids were applied at a 1:3 ratio while maintaining a total omega-3 fatty acid concentration of 25 μM, thereby ensuring comparability between individual and combined treatment conditions in terms of overall fatty acid exposure. Similarly to the DKK1 procedure, these treatments were applied after a 10-min incubation with the respective individual EC_25_ concentrations. As previously reported, sterile water and 0.3% ethanol were used as the vehicles for MPH and ω-3 PUFAs, respectively (Walter et al., 2025).

### 2.5 Data and statistical analysis

Outliers from the Wnt-reporter assays were detected using the interquartile range method across the six replicates from two independent experiments, as previously described (Yde Ohki et al., 2023b). The list of cell lines included in each analysis and the number of independent experiments performed for each scenario are provided in Supplementary Table 2.

All statistical analyses were conducted in R (version 4.4.2), except for the correlation analyses, which were performed and visualized in SPSS (version 29.0.0). Correlations between ADHD-PRS and EC_50_ or IC_50_ values were examined using Spearman's correlation. All other graphs were generated in GraphPad Prism (version 10.6.1).

EC_50_ and IC_50_ values were compared between ADHD- and control-derived NSC lines using Mann–Whitney U tests (wilcox.test) (Supplementary Table 3).

For the Wnt reporter experiments following treatment with MPH, PUFAs and combined treatments, normalized Wnt activity was analyzed using linear mixed-effects models (LMMs) fitted with lmer from *lme4* and *lmerTest*. Three separate LMMs were fitted: one for EPA (Vehicle, 25 μM, 50 μM), one for DHA (Vehicle, 25 μM, 50 μM), and one combined model including all six conditions (Vehicle DHA + EPA, DHA + EPA, Vehicle DHA + MPH, DHA + MPH, Vehicle MPH, MPH). Fixed effects were diagnostic group, condition (treatment type or concentrations), and their interaction. To account for the experimental hierarchy, random intercepts were included for cell line and for plate nested within cell line *(1 | cell_line + 1 | cell_line:plate_id)*. Type III F-tests for fixed effects were obtained with Satterthwaite-approximated degrees of freedom (anova from *lmerTest*). Model assumptions were evaluated using residual-vs-fitted and Q–Q plots.

Estimated marginal means (EMMs) and pairwise contrasts were computed using *emmeans*. In the EPA and DHA models, pairwise comparisons of the three conditions within each diagnostic group were performed with Tukey adjustment for the three condition levels.

In the combined six-condition model, the primary planned contrasts compared each active treatment with its corresponding vehicle within each group and used a Sidak correction. Additional pairwise comparisons among conditions within each group were examined with Tukey adjustment. Between-group comparisons within each condition represent single comparisons and their p-values were not further adjusted for multiplicity.

Standardized effect sizes (Hedges’ g) for model-based contrasts were computed from the residual standard deviation of each model and interpreted using conventional benchmarks (small ≈ 0.2, medium ≈ 0.5, large ≥0.8).

Supplementary Table 4 presents the effect sizes for various pairwise comparisons between the aADHD and aControl groups using Hedge's *g* (appropriate given the small sample size of 5 per group)*.* Supplementary Table 5 reports effect sizes between treated conditions within each group (treated *vs.* vehicle, EPA 25 μM vs. 50 μM and DHA 25 μM vs. 50 μM).

## 3 Results

**Fig. 1:**
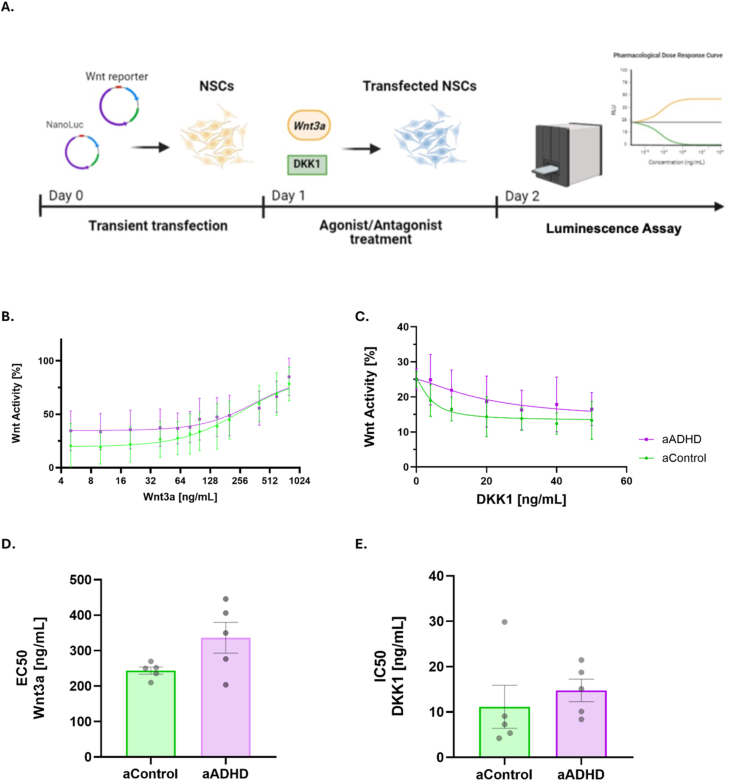
**Assessment of basal Wnt activity in NSCs after Wnt3a and DKK1 treatments.** A) Schematic representation of the Wnt reporter assays using NSCs derived from adult ADHD patients (aADHD) and healthy controls (aControl). B) Ascending dose‒response curves for all the cell lines following treatment with the Wnt-agonist Wnt3a. C) Descending dose‒response curves for all the cell lines after treatment with the Wnt antagonist DKK1. D) EC_50_ values of Wnt3a in the aADHD and aControl groups (N = 5 per group). E) IC_50_ values after DKK1 treatment in the aADHD and aControl cell lines (N = 5 per group). Each dot represents the average of two independent experiments from each individual cell line. The bar charts represent mean ± SEM.

Biologically characterized NSCs from adult ADHD patients and healthy controls (aControl) (Supplementary Fig. 2) were subjected to Wnt-reporter assays (Fig. 1A).

After treatment with increasing concentrations of the Wnt-agonist Wnt3a and the Wnt-antagonist DKK1, dose‒response curves were generated for each group (Fig. 1B and 1C). Plotting dose‒response curves for each cell line (Supplementary Figs. 3 and 4) allowed us to determine individual EC_50_ and IC_50_ values by setting the highest mean RLU value as 100% in the case of EC_50_ calculations and the lowest mean RLUs as bottom values when IC_50_ were to be determined, as previously described (Yde Ohki et al., 2023b).

There were no statistically significant group differences in EC_50_ values for Wnt3a or IC_50_ values for DKK1 between the aADHD and aControl NSC lines. However, for EC_50_, the effect size calculations showed large (Hedge's *g* ≈ 1.31) and medium magnitude for EC_50_ and IC_50_, respectively (Hedge's *g* ≈ 0.43). Overall, these findings indicate no robust group-level shift in dose–response parameters (Fig. 1D and 1E).

Given the heterogeneity of our aADHD group, we aimed to identify possible correlations between genetic predisposition to ADHD (ADHD-PRS) and Wnt3a EC_50_ or DKK1 IC_50_ values. Whereas EC_50_ values showed no association with ADHD-PRS, IC_50_ values after DKK1 administration correlated significantly and positively with ADHD-PRS (Supplementary Fig. 5A and 5B). No significant correlations were observed when the aADHD and aControl groups were analyzed separately (Supplementary Fig. 5C–F).

To investigate whether age could influence basal Wnt activity, we performed a correlation analysis between EC_50_/IC_50_ values and age of our adult participants including also the children and adolescents from Walter et al., 2024). Whereas a significant positive correlation between EC_50_ and age was found (Spearman's ρ = 0.556; ∗*p* = 0.011) (Supplementary Fig. 6A), only a non-statistically significant tendency of negative correlation was observed for IC_50_ (Spearman's ρ = 0.164; *p* = 0.469) (Supplementary Fig. 6B). When only ADHD cell lines (from adults and pediatric patients) were analyzed, a significant positive correlation between age and EC_50_ was found only in the ADHD group (Supplementary Fig. 6C), but not in controls (Supplementary Fig. 6E). Both groups, when analyzed separately, showed the same pattern of response in the case of IC_50_ *versus* age (Supplementary Fig. 6D and 6F).

Similarly, we tested whether excluding females from the aADHD and aControl groups would result in group differences in terms of EC_50_/IC_50_ comparisons. A nominal increase in EC_50_ concentrations (and therefore, decrease in Wnt activity) was seen for the aADHD group (Mann-Whitney, ∗*p* = 0.057) (Supplementary Fig. 7A), as opposed to the initial context in which both sexes were included to the analysis (Fig. 1). However, including only males to the analysis has not changed the previously observed results for IC_50_ (Supplementary Fig. 7B).

**Fig. 2:**
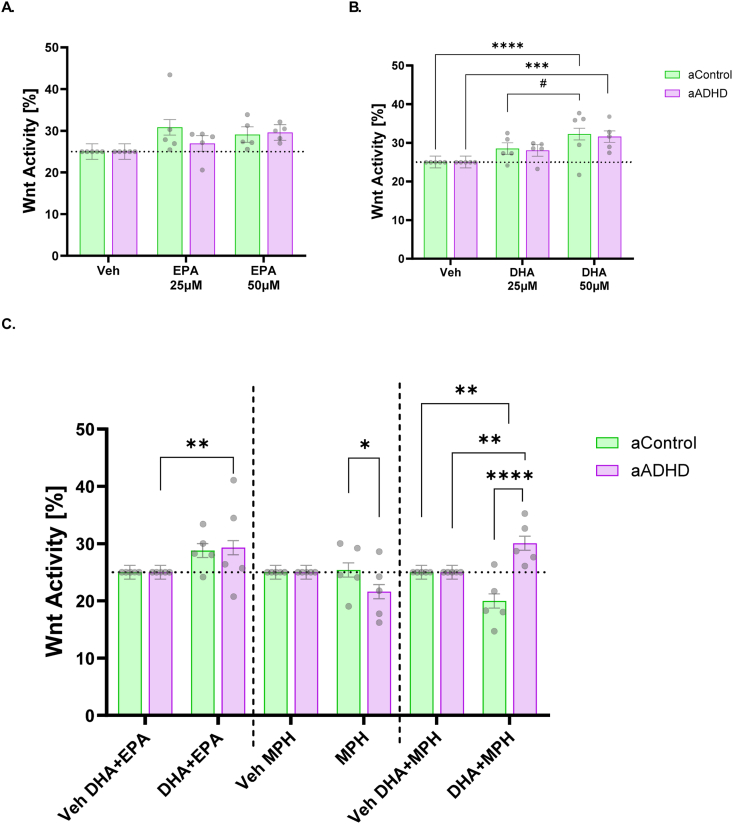
**Wnt-related effects of EPA, DHA, MPH and combined DHA + EPA and DHA + MPH treatments in aADHD and aControl NSCs.** A) Effects of EPA at 25 and 50 μM on normalized Wnt activity in NSCs from adult ADHD (aADHD) and adult control (aControl) donors. B) Effects of DHA at 25 and 50 μM on normalized Wnt activity of both groups. C) Combined-condition model including three treatments and their respective vehicles: DHA + EPA (8.3 μM + 16.7 μM; 1:3 ratio); MPH (10 nM); DHA + MPH (25 μM + 10 nM). Bars show estimated marginal means (EMMs ± SE) from the mixed-effects model. Each dot represents the mean normalized Wnt activity for one NSC line (averaged across replicate measurements). In all graphs from Fig. 2, Wnt activity values were normalized to 25%, as NSCs were pre-treated with their individual EC25 concentrations of Wnt3a prior to the addition of DHA, EPA, and MPH. Asterisks indicate *p* < 0.05 (after the appropriate multiplicity correction).

We next investigated whether ω-3 PUFAs had Wnt-related modulatory effects in these cell lines. For EPA, the LMM showed no main effect of diagnostic group (*F*(1, 174) = 0.54, *p* = 0.46) and no group × concentration interaction (*F*(2, 174) = 0.83, *p* = 0.44). However, there was a significant main effect of concentration (*F*(2, 174) = 3.22, ∗*p* = 0.043). Tukey-adjusted pairwise contrasts within each group showed no statistically significant differences between individual EPA doses and vehicle (all adjusted p ≥ 0.07) (Fig. 2A).

Regarding DHA treatments, a significant main effect of DHA concentration was found (*F*(2, 156) = 17.07, ∗∗∗∗*p* = 2.00 x 10^−7^), while no significant differences were observed for group (*F*(1, 8) = 0.05, *p* = 0.82) or for the group × concentration interaction (*F*(2, 156) = 0.04, *p* = 0.96). DHA at 50 μM induced a significant (after Tukey correction) upregulation of Wnt activity within both the aADHD group (EMM: 31.6 *vs.* 25.0; estimate = −6.56, SE = 1.67, t(156) = −3.93, ∗∗∗*p* = 0.0004; Hedge's *g* ≈ 1.01, large) and the aControl group (EMM: 32.2 *vs.* 25.0; estimate = −7.21, SE = 1.67, t(156) = −4.32, ∗∗∗∗*p* = 0.0001; Hedge's *g* ≈ 1.11, large). Moreover, no statistically significant differences in Wnt activity were observed in the aControl group between 25 and 50 μM (EMM: 28.5 *vs.* 32.2; estimate = −3.74, SE = 1.67, t(156) = −2.243, *p* = 0.067; Hedge's g ≈ 0.58, medium) (Fig. 2B).

Our next goal was to evaluate how MPH and combined treatment conditions (DHA + EPA, DHA + MPH) modulate Wnt signaling across aADHD and aControl NSC lines. The combined six-condition LMM showed a significant main effect of condition (*F*(5, 336.12) = 5.07, ∗∗∗∗*p* = 1.71 × 10^−4^) and a significant group × condition interaction (*F*(5, 336.12) = 7.88, ∗∗∗∗*p* = 4.96 × 10^−7^), indicating differential treatment responses in aADHD *vs.* aControl lines (Fig. 2C).

Within aADHD lines, both combined treatments increased Wnt activity relative to their respective vehicles: MPH (21.6 *vs.* 25.0; estimate = −3.39, SE = 1.66, t(326) = −2.05, *p* = 0.12, Hedge's *g*≈ 0.53, medium), DHA + EPA (29.3 *vs.* 25.0; estimate = 4.30, SE = 1.66, t(326) = 2.59, ∗*p* = 0.029, Hedge's *g*≈ 0.67, medium), and DHA + MPH (30.1 *vs.* 25.0; estimate = 5.06, SE = 1.64, t(326) = 3.08, ∗∗*p* = 0.0067; Hedge's *g* ≈ 0.79, medium) after Sidak correction. Within aControl lines, MPH (25.4 *vs.* 25.0; estimate = 0.39, SE = 1.66, t(326) = 0.23, *p* = 0.99, Hedge's *g*≈ 0.06, negligible), DHA + EPA minimally increased Wnt activity compared to its vehicle (28.8 *vs,* 25.0; estimate = 3.78, SE = 1.64, t(326) = 2.30, *p* = 0.065; Hedge's *g*≈ 0.59, medium), whereas DHA + MPH significantly decreased Wnt activity relative to its vehicle (20.0 vs. 25.0; estimate = −5.03, SE = 1.66, t(326) = −3.03, ∗∗*p* = 0.0079; Hedge's *g* ≈ 0.79, medium) after Sidak correction (Fig. 2C).

Group-wise EMMs revealed a pronounced divergence in Wnt activity under the DHA + MPH combination: aADHD lines showed significantly higher Wnt activity than controls (EMMs: 30.1 *vs.* 20.0; estimate = 10.09, SE = 1.73, t(113) = 5.82, ∗∗∗∗*p* < 0.0001, Hedge's *g* ≈ 1.6, large). Moreover, a statistically significant reduction in Wnt activity in aADHD relative to aControl was found under MPH alone (EMMs: 21.6 *vs.* 25.4; estimate = −3.78, SE = 1.75, t(116) = −2.163, ∗*p* = 0.033, Hedge's *g* ≈ 0.6, medium) (Fig. 2C).

Effect sizes for all relevant comparison are provided in Supplementary Tables 4 and 5

## 4 Discussion

The canonical Wnt signaling is essential for maintaining cellular events throughout the lifespan. Defects in its activity may lead to distinct cellular phenotypes that could ultimately be associated with poor clinical outcomes, as already observed in iPSC models from other neurodevelopmental disorders (e.g., autism spectrum disorders, Pitt-Hopkins Syndrome, and schizophrenia (Papes et al., 2022; Marchetto et al., 2017; Sawada et al., 2020)).

We generated NSCs from adult ADHD and control subjects and quantified their Wnt activity using the same protocols previously described (Walter et al., 2024; Yde Ohki et al., 2023b). However, the results in iPSC-derived NSCs from adults do not fully resemble the findings from ADHD children and adolescents. In the current study, we observed no differences in basal Wnt activity between cell lines derived from adult ADHD patients and controls. Several factors may account for this discrepancy. First, differences in the recruitment process for our adult participants may have introduced variability. Whereas the presence of psychiatric comorbidities was an exclusion criterion in the pediatric cohort (Yde Ohki et al., 2021, 2023c, 2024; Grossmann et al., 2021), the adult individuals could present comorbidities, for example Major Depressive Disorder (MDD). Secondly, the previously analyzed NSCs were derived from pediatric ADHD patients who clinically responded to MPH (Walter et al., 2024), whereas responsiveness to treatment was more diverse for the adult individuals. Thirdly, in the pediatric study, patients and controls were stratified based on their genetic predisposition to ADHD, whereas in adults, environmental factors accumulated across the lifespan may have also influenced the phenotype. Therefore, a more homogeneous stratification of the aADHD group may be crucial to reveal more robust effects.

Following the same rationale, the aADHD and aControl groups in the present study were not balanced with respect to sex and genetic predisposition to ADHD, unlike the previous cohort of children and adolescents (Walter et al., 2024). A modest change in the statistical *p*-value was seen in the EC_50_ analysis after removing results from female individuals, which might potentially suggest that sex might influence the levels of Wnt activity. However, these results must be cautiously interpreted, given our current small sample size and absence of statistical significance.

Given that Wnt-related parameters (specifically the protein expression of active β-catenin and inactive GSK3β) significantly correlated with ADHD-PRS in our previous investigations (Walter et al., 2024), it is likely that genetic predisposition to ADHD plays a role in Wnt activity. Thus, to observe clearer group-level patterns, it may be essential to stratify study participants not only by their diagnosis but also by their genetic liability to ADHD. In fact, whereas correlations between ADHD-PRS and Wnt3a EC_50_ values in NSCs from children and adolescents tended to be negative (Walter et al., 2024), the adult-derived NSCs in the current study show a tendency toward positive correlations. For DKK1, we observed a significant positive correlation between its IC_50_ values and ADHD-PRS, despite the absence of significant differences between aControl and aADHD. This relationship not only reinforces the need for PRS-based stratification within ADHD cohorts but also aligns with findings from our pediatric ADHD sample (Walter et al., 2024), as the correlation suggests that adult subjects with high genetic liability generate NSCs with higher IC_50_ values and therefore higher Wnt activity, as we found in pediatric ADHD donors. By performing a correlation analysis between its IC_50_ values and ADHD-PRS in aADHD and aControl lines separately, we observed the same pattern of response, which might indicate that this response is not diagnosis-specific, unlike the correlation between EC_50_ values and ADHD-PRS. However, this has to be cautiously interpreted due to the small sample size.

We recognize that certain technical limitations may account for the discrepancies between the current and previous results. In the current study, only one clone per donor was used, whereas two clones per donor were used in our earlier work (Walter et al., 2024), which may have impacted the statistical power of the analyses. Moreover, it is important to note that the somatic cell type used for iPSC generation differed between cohorts: the adult iPSCs were generated from fibroblasts, while whereas the pediatric iPSCs were derived from Peripheral Blood Mononuclear Cells (PBMCs) and keratinocytes (Walter et al., 2024).

The cell type of origin for iPSC reprogramming may influence neural differentiation, as epigenetic memory can persist despite reprogramming (Kim et al., 2010; Hargus et al., 2014). Although the iPSC community largely agrees that reprogramming somatic cells into iPSCs fully erases epigenetic markers (Polo et al., 2012), this assumption remains a matter of debate (Ohi et al., 2011; Khan et al., 2022). In this context, epigenetic signatures acquired accumulated throughout the lifespan in adult donors may not be entirely eliminated during reprogramming, potentially resulting in different Wnt-related cellular responses in NSCs derived from pediatric and adult patients. This interpretation aligns with evidence that epigenetic factors have regulatory roles in the Wnt pathway, particularly in cancer (Sharma et al., 2021; Urakami et al., 2006). It is possible that this differential modulation in the Wnt pathway is related to its involvement in other functions during adulthood (e.g., homeostatic maintenance) rather than neurodevelopmental processes commonly present in embryogenesis and childhood (Grainger and Willert, 2018). Our findings further support the hypothesis of persistent epigenetic influences, as demonstrated by the significant correlation between Wnt3a EC_50_ values and age in both ADHD patients and controls, including data from Walter and colleagues (Walter et al., 2024). This significant correlation persists when only aADHD cell lines were analyzed, but not aControl NSCs, which might suggest a potential impact of the donors’ age to the Wnt activity specifically in ADHD. While definitive conclusions cannot yet be drawn regarding differences between findings from ADHD children and adults, these considerations provide important hypotheses for future work.

Although no group differences in basal Wnt activity were observed, we observed promising effects of DHA, but not EPA, on Wnt activity. Although the divergent response patterns elicited by EPA and DHA do not align with our initial hypothesis, this outcome is not unexpected, as these PUFAs may play distinct roles in different cellular mechanisms (Pham et al., 2018; Gustafsson et al., 2010; Ganesan et al., 2012). In our study, DHA at a concentration of 50 μM upregulated Wnt activity in both aControl and aADHD cell lines, consistent with the findings of Zhao et al. in human iPSC-derived neural progenitor cells (Zhao et al., 2019). In contrast, our current results do not replicate our previous observations that EPA at the same concentration (50 μM) increased Wnt activity in NSCs derived from children and adolescents with ADHD (Walter et al., 2025). Neither 25 μM nor 50 μM EPA modulated Wnt activity in the NSCs from adult subjects. Similarly, combining DHA (8.3 μM) and EPA (16.7 μM) at a 1:3 ratio enhanced Wnt activity in both groups. While these findings suggest that other EPA doses or ratios may merit further investigation, they may also indicate more general beneficial effects of ω-3 PUFAs. This interpretation is in line with a previous report from Bos and collaborators, who showed that EPA/DHA supplementation improved attention in both children with and without ADHD, with no differences between the groups (Bos et al., 2016).

Moreover, several differences remain between findings from NSCs derived from pediatric versus adult ADHD patients. For instance, while 10 nM MPH enhanced Wnt activity in NSCs from children and adolescents who were clinically responsive to MPH treatment (Walter et al., 2025; Yde Ohki et al., 2025), we observed that MPH induced a downregulation of Wnt activity in aADHD NSCs. This discrepancy may reflect developmental differences in MPH response between children and adolescents and adults. A randomized clinical trial found greater thalamic and striatal blood flow in children than in adults with ADHD after 16-weeks of MPH treatment (Schrantee et al., 2016). Similarly, differential connectivity patterns in the thalamus and striatum has been observed between children and adults with ADHD following acute MPH administration (Kaiser et al., 2022). Furthermore, a systematic review and meta-analysis indicated that MPH is generally less tolerable and effective in adults compared with children (Cortese et al., 2018).

Previous evidence has demonstrated links between beneficial effects on cognition*,* ω-3 supplementation, and activation of the canonical Wnt pathway. *In vivo*, ω-3 administration has been shown to increase the protein expression of β-catenin in brains of animal models of pre-eclampsia. Furthermore, β-catenin levels were positively correlated with improvements in cognitive functions indicative of diminished anxiety-like behavior (ShamsEldeen et al., 2021). In humans, ω-3 supplementation has also been associated with beneficial effects. For instance, high-dose EPA improved attention and vigilance in individuals with ADHD (Chang et al., 2019). However, our results must be interpreted with caution, as the increases in Wnt activity observed after DHA in our NSCs cannot yet be directly translated into clinical improvements. The combination of DHA (25 μM) and MPH (10 nM) led to a group-specific increase in Wnt activity in ADHD NSCs, while MPH alone downregulated Wnt activity in this group, and DHA alone did not display any modulatory effects in any of the groups. As MPH is a selective dopamine transporter blocker, it is plausible that this drug modulates the Wnt activity in a dopamine-dependent manner, as the activation of the dopamine D2 receptor (D2R)-mediating signaling switch on the canonical Wnt pathway occurs through GSK3β inactivation (Han et al., 2019). On the other hand, it has been shown that DHA may act as an allosteric modulator of D2R, facilitating ligand binding and enhancing its activity (Jobin et al., 2023). Thus, one hypothesis is that a possible increase in D2R activity mediated by DHA is potentiated by the elevated MPH-induced presence of dopamine. As a result, the Wnt activity would also be enhanced. However, more profound investigations are required to draw conclusions about exact underlying mechanisms and the effects of these treatments in these cell lines in terms of cellular phenotypes (e.g., proliferation and differentiation processes).

Additionally, further studies would have to be conducted to understand whether there are any clinical implications of the decreased Wnt activity promoted by the combination of DHA and MPH in healthy controls and increased activity in the ADHD group. Our findings align with clinical trials that reported that ω-3 (EPA + DHA) supplementation alongside MPH reduced symptom scores in patients with ADHD (Barragán et al., 2017; Checa-Ros et al., 2019). In the report from Barragán and collaborators (Barragán et al., 2017), hyperactivity/impulsivity scores and total ADHD scores were significantly reduced after the combination between ω-3/6 (EPA + DHA + ω-6) and MPH in comparison to the treatment with only PUFAs, but not impulsivity (Barragán et al., 2017).

If future studies are able to confirm that Wnt activation positively correlates with improved ADHD symptomatology, our results would contradict the findings from Checa-Ros and colleagues., who showed that adding DHA as an adjuvant therapy to MPH does not affect ADHD clinical scores (Mohammadzadeh et al., 2019). Nevertheless, given the evidence in the literature, our results warrant further investigation of the relationship between Wnt modulation and clinical improvements by an essential non-pharmacological approach (*i.e.,* ω-3 PUFAs) in adult ADHD.

## Author contributions

CMYO performed experiments, conducted data analysis and wrote the first draft of the manuscript. NW performed experiments, generated graphs and extracted EC50/IC50 values and wrote the first draft of the manuscript. LD, LDC and RL performed experiments, generated graphs and extracted EC50/IC50 values. RVMN and ZS performed experiments and data analysis on iPSCs. LS conducted data analysis, PRS calculations and reviewed the manuscript. SW reviewed the manuscript. SKS and EG conceptualized and supervised the project and reviewed the manuscript.

## Funding

This project was financed by the Waterloo Foundation (reference number 2462/4548).

## Conflict of interest

SW has received royalties within the past five years from Thieme, Hogrefe, Kohlhammer, Springer, and Beltz. In 2023, she received an honorarium as a speaker from Takeda. Her work has been supported in recent years by the Swiss National Science Foundation (SNF), various EU FP7 programs, HSM (Hochspezialisierte Medizin) of the Canton of Zurich, BfArM Germany, ZInEP, the Hartmann Müller Foundation, the Olga Mayenfisch Foundation, the Gertrud Thalmann Foundation, Vontobel, Unicentia, the Erika Schwarz Foundation, Heuberg Foundation, the Swiss Federal Office of Public Health (BAG), Gesundheitsförderung Schweiz, and Horizon Europe. Information regarding her outside professional activities and interests is publicly available on the University of Zurich website: www.uzh.ch/prof/ssl-dir/interessenbindungen/client/web/

SKS has received speaker's honoraria from Takeda, Janssen and Medice Arzneimittel Pütter GmbH in the past 3 years.

EG's work has been supported in recent years by the Hartmann Müller Stiftung für Medizinische Forschung, Waterloo Foundation, McGill and Neuroscience Center Zurich collaborative grant, Personalized Health and Related Technologies (PHRT), and MEDICE Arzneimittel Pütter GmbH & Co.

The other authors do not have any conflicts of interest to disclose.
