# Supplementary material for: The effects of omega-3 fatty acids on Wnt activity in iPSC-derived neural stem cells from adult ADHD patients
Source: Neurosci Appl. 2026 May 2;5:107005. doi: 10.1016/j.nsa.2026.107005 (PMC13185858; doi:10.1016/j.nsa.2026.107005)
Supplement: Multimedia component 1 [file mmc1.pdf]

The effects of omega-3 fatty acids on Wnt activity in iPSC-derived neural stem cells from adult ADHD patients

Cristine M. Yde Ohki[1*], Natalie M. Walter[1*], Louisa C. Dury[1*], Letizia Del Campana[1], Romane Lasmarrigues[1], Rhiannon V. McNeill[2], Zora Schickardt[2], Lukasz Smigielski[1], Susanne Walitza[1,3,4], Sarah Kittel-Schneider[2,5,6*], Edna Grünblatt[1,3,4*]

[1] Department of Child and Adolescent Psychiatry and Psychotherapy, Translational Molecular Psychiatry, University Hospital of Psychiatry Zurich, University of Zurich, Wagistrasse 12, 8952, Schlieren, Switzerland

[2] Department of Psychiatry, Psychosomatics and Psychotherapy, Center of Mental Health, University of Würzburg, Würzburg, Germany.

[3] Neuroscience Center Zurich, University of Zurich and the ETH Zurich, Winterthurerstrasse 11, 8057, Zurich, Switzerland

[4] Zurich Center for Integrative Human Physiology, University of Zurich, Winterthurerstrasse 11, 8057, Zurich, Switzerland

[5] Department of Psychiatry and Neurobehavioural Science, University College Cork, Cork, Ireland

[6] APC Microbiome, University College Cork, Cork, Ireland

* First/last authors contributed equally.

Supplementary Information

Supplementary Table 1. Demographic information for the subjects included in this study.

Cell line	Diagnosis	Age	Sex	ADHD-PRS (z-score)	iPSC QC can be found on
CTL1	aControl	25	Male	0.442	Suppl. Fig. 1
CTL2	aControl	28	Male	-1.674	McNeill et al. 2025
CTL3	aControl	45	Male	0.550	Suppl. Fig. 1
CTL4	aControl	27	Female	0.051	McNeill et al. 2025

CTL5	aControl	39	Female	-0.209	Palladino et al. 2020
ADHD1	aADHD	53	Male	-0.133	Suppl. Fig. 1
ADHD2	aADHD	39	Male	-1.696	McNeill et al. 2023
ADHD3	aADHD	27	Male	1.109	Suppl. Fig. 1
ADHD4	aADHD/MDD	46	Male	1.175	Suppl. Fig. 1
ADHD5	aADHD	30	Female	0.385	Palladino et al. 2020

Supplementary Table 2. List of all Wnt-reporter analyses conducted in this study.

Cell line	Readout						
	Calculation of EC_{50} values after Wnt3a	Calculation of IC_{50} values after DKK1	Determination of Wnt activity after MPH 10 nM	Determination of Wnt activity after EPA (25 and 50 µM)	Determination of Wnt activity after DHA (25 and 50 µM)	Determination of Wnt activity after MPH 10 nM + DHA 25 µM	Determination of Wnt activity after EPA 16.7 µM + DHA 8.3 µM
CTL1	✓✓	✓✓	✓✓	✓✓	✓✓	✓✓	✓✓
CTL2	✓✓	✓✓	✓✓	✓✓	✓✓	✓✓	✓✓
CTL3	✓✓	✓✓	✓✓	✓✓	✓✓	✓✓	✓✓
CTL4	✓✓	✓✓	✓✓	✓✓	✓✓	✓✓	✓✓
CTL5	✓✓	✓✓	✓✓	✓✓	✓✓	✓✓	✓✓
ADHD1	✓✓	✓✓	✓✓	✓✓	✓✓	✓✓	✓✓
ADHD2	✓✓	✓✓	✓✓	✓✓	✓✓	✓✓	✓✓
ADHD3	✓✓	✓✓	✓✓	✓✓	✓✓	✓✓	✓✓
ADHD4	✓✓	✓✓	✓✓	✓✓	✓✓	✓✓	✓✓
ADHD5	✓✓	✓✓	✓✓	✓✓	✓✓	✓✓	✓✓

Each check mark (✓) equals to one independent experiment.

Supplementary Table 3. EC_{50} and IC_{50} values after treatment with Wnt3a and DKK1, respectively.

Cell line	Diagnosis	Wnt3a's EC_{50} (ng/mL)	DKK1's IC_{50} (ng/mL)
CTL1	Healthy adult	209.7	29.8
CTL2	Healthy adult	269.9	7.279
CTL3	Healthy adult	249.9	9.09
CTL4	Healthy adult	235.7	5.34
CTL5	Healthy adult	251.8	4.213
ADHD1	Adult ADHD	349.9	10.11
ADHD2	Adult ADHD	276.3	8.37
ADHD3	Adult ADHD	405.9	21.44
ADHD4	Adult ADHD	445.7	15.13
ADHD5	Adult ADHD	203.7	18.74

Supplementary Table 4. Effect sizes (Hedge's *g*) for pairwise comparisons between the aADHD and aControl groups.

**Parameter**	**Hedge's *g***	**Magnitude**
**Wnt3a EC_{50}**	**1.311**	**Large**
DKK1 IC_{50}	***0.426***	***Medium***
EPA 25µM	0.373	Small
EPA 50µM	0.049	Negligible
DHA 25µM	0.073	Negligible
DHA 50µM	0.098	Negligible
EPA + DHA	0.081	Negligible
MPH 10nM	***0.59***	***Medium***
**MPH + DHA**	**1.574**	**Large**

Supplementary Table 5. Effect sizes (Hedge's *g*) for pairwise comparisons between treated conditions following PUFAs, MPH and combined treatments.

**Parameter**	**Group**	**Hedge's *g***	**Magnitude**
EPA 25µM x Vehicle	aADHD	0.188	Negligible
	aControl	***0.564***	***Medium***
EPA 50µM x Vehicle	aADHD	0.443	Small
	aControl	0.394	Small
EPA 25µM x EPA 50µM	aADHD	0.255	Small
	aControl	0.171	Negligible
DHA 25µM x Vehicle	aADHD	0.46	Small
	aControl	***0.535***	***Medium***
DHA 50µM x Vehicle	**aADHD**	**1.01**	**Large**
	**aControl**	**1.111**	**Large**
DHA 25µM x DHA 50µM	***aADHD***	***0.551***	***Medium***
	aControl	***0.576***	***Medium***
EPA + DHA x Vehicle	***aADHD***	***0.673***	***Medium***
	aControl	***0.592***	***Medium***
MPH 10nM x Vehicle	***aADHD***	***0.601***	***Medium***
	aControl	0.068	Negligible
MPH + DHA x Vehicle	***aADHD***	***0.763***	***Medium***
	aControl	***0.788***	***Medium***

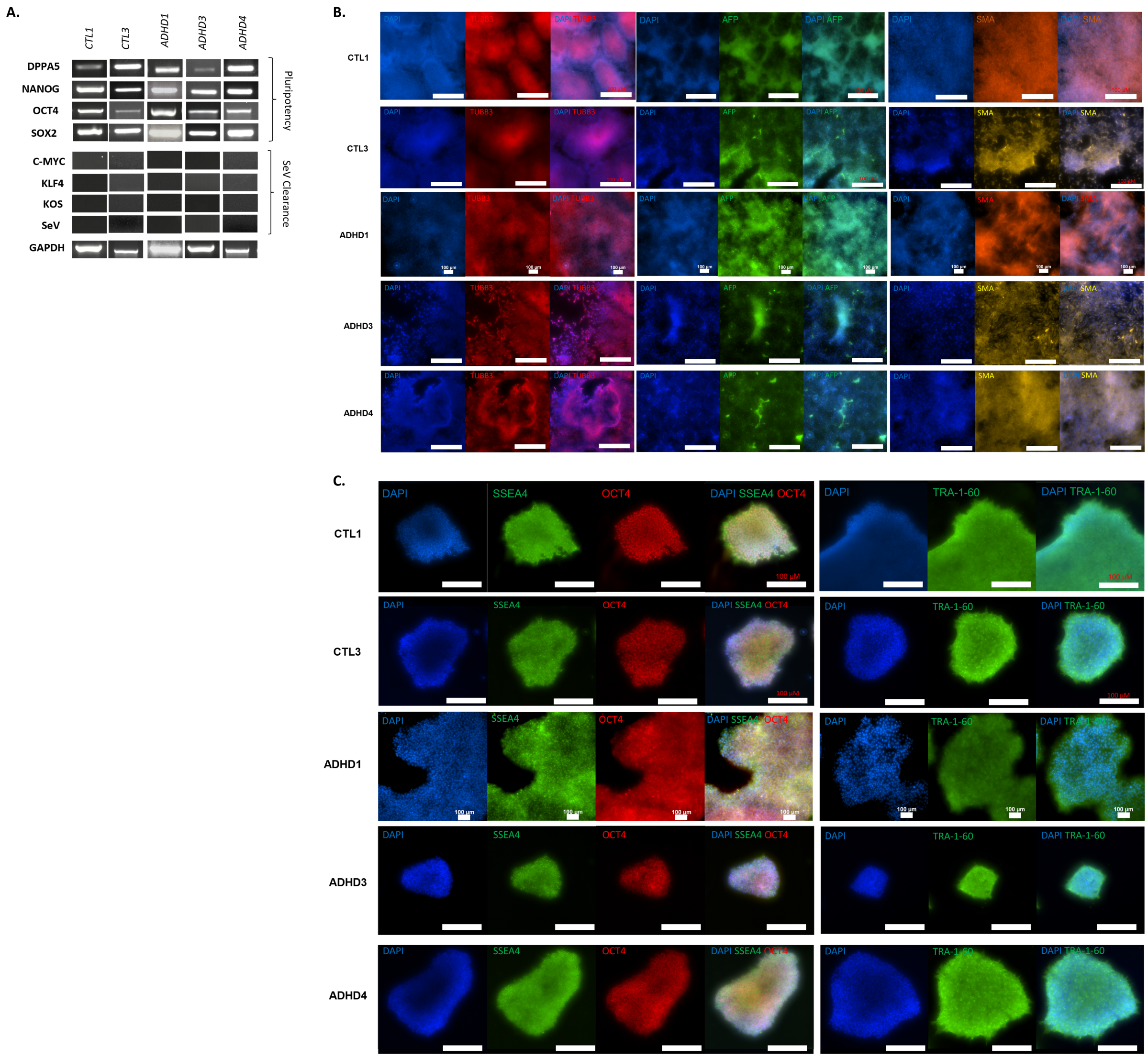

Supplementary Figure 1. Quality control of iPSCs derived from adult ADHD patients and matched healthy controls. A) RT-qPCR amplicon bands demonstrate positive gene expression of pluripotency genes (*DPPA5*, *NANOG*, *OCT4* and *SOX2*) and absence of Sendai virus (SeV)-related genes. *GAPDH* was used as reference gene. Individual bands were cropped from the original images for better visualization. B) Immunocytochemistry (ICC) was used to confirm the ability of differentiation into the three germ layers (endoderm, mesoderm and ectoderm) through positive expression of AFP, SMA and TUBB3, respectively, in iPSCs CTL1, CTL3, ADHD1, ADHD3 and ADHD4. Scale bar: 100 μm. C) The same technique was applied to demonstrate the positive protein expression of pluripotency markers SSEA4, OCT4 and TRA-1-60. Scale bar: 100 μm.

A.

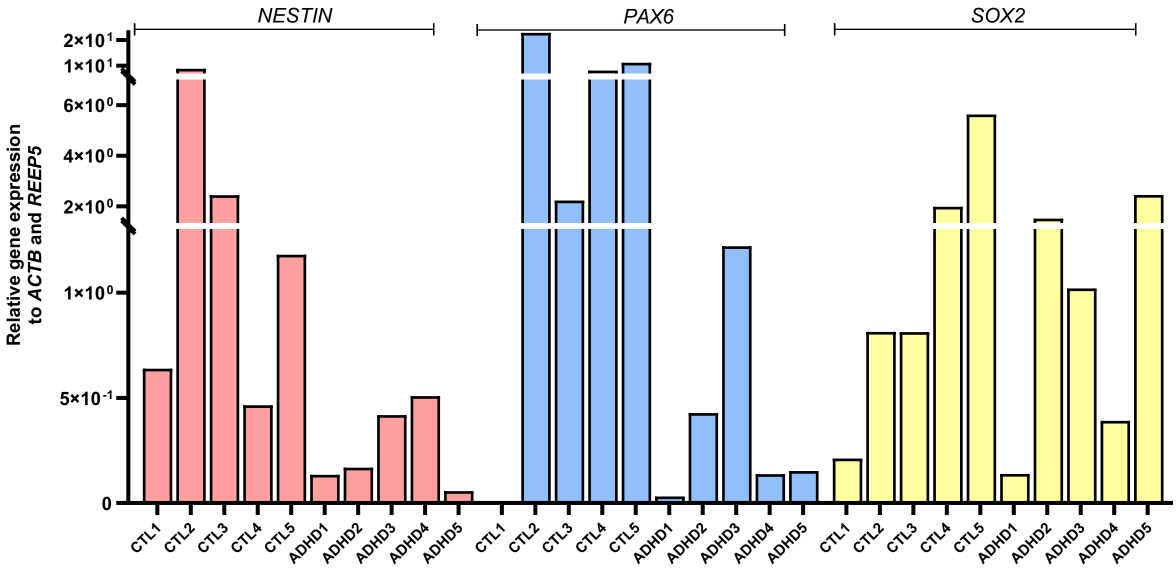

B.

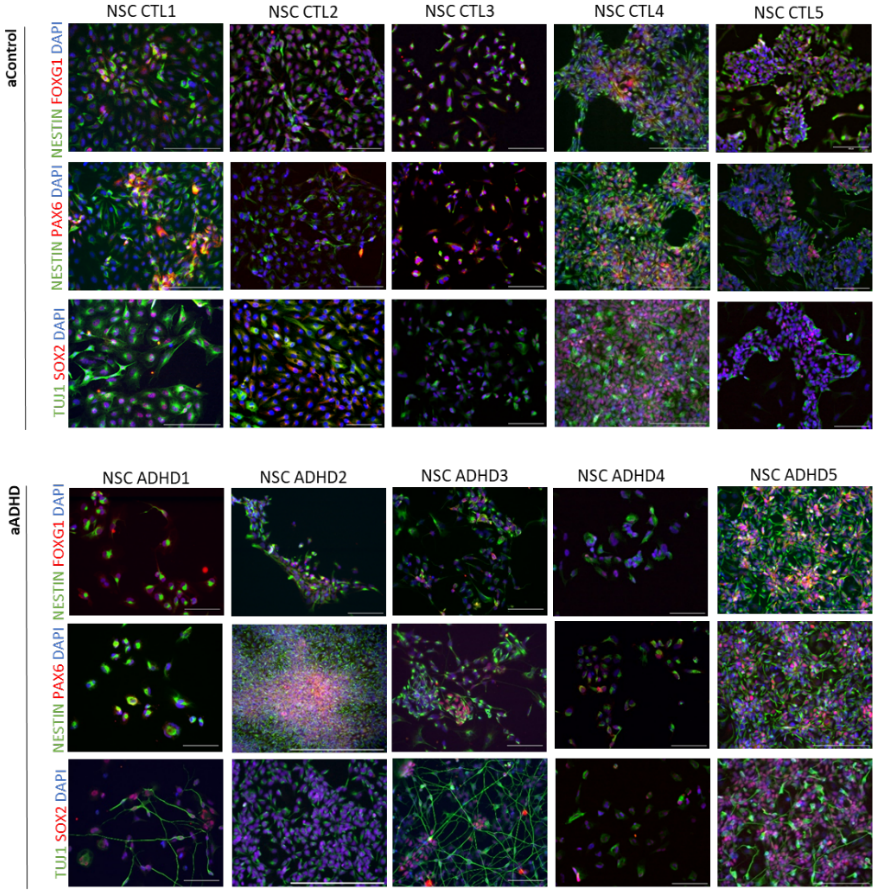

Supplementary Figure 2. Quality control of iPSC-derived NSCs. A) Heatmap showing the relative gene expression of *SOX2*, *Nestin* and *PAX6* after RT-qPCR. *ACTB* and *REEP5* served as reference genes. B) Representative immunocytochemistry images from all cell lines included in this study showing positive protein expression of classical NSC markers (FOXG1, NESTIN, SOX2, PAX6 and TUBB3). Scale bar: 100 µm. aControl: adult control; aADHD: adult ADHD.

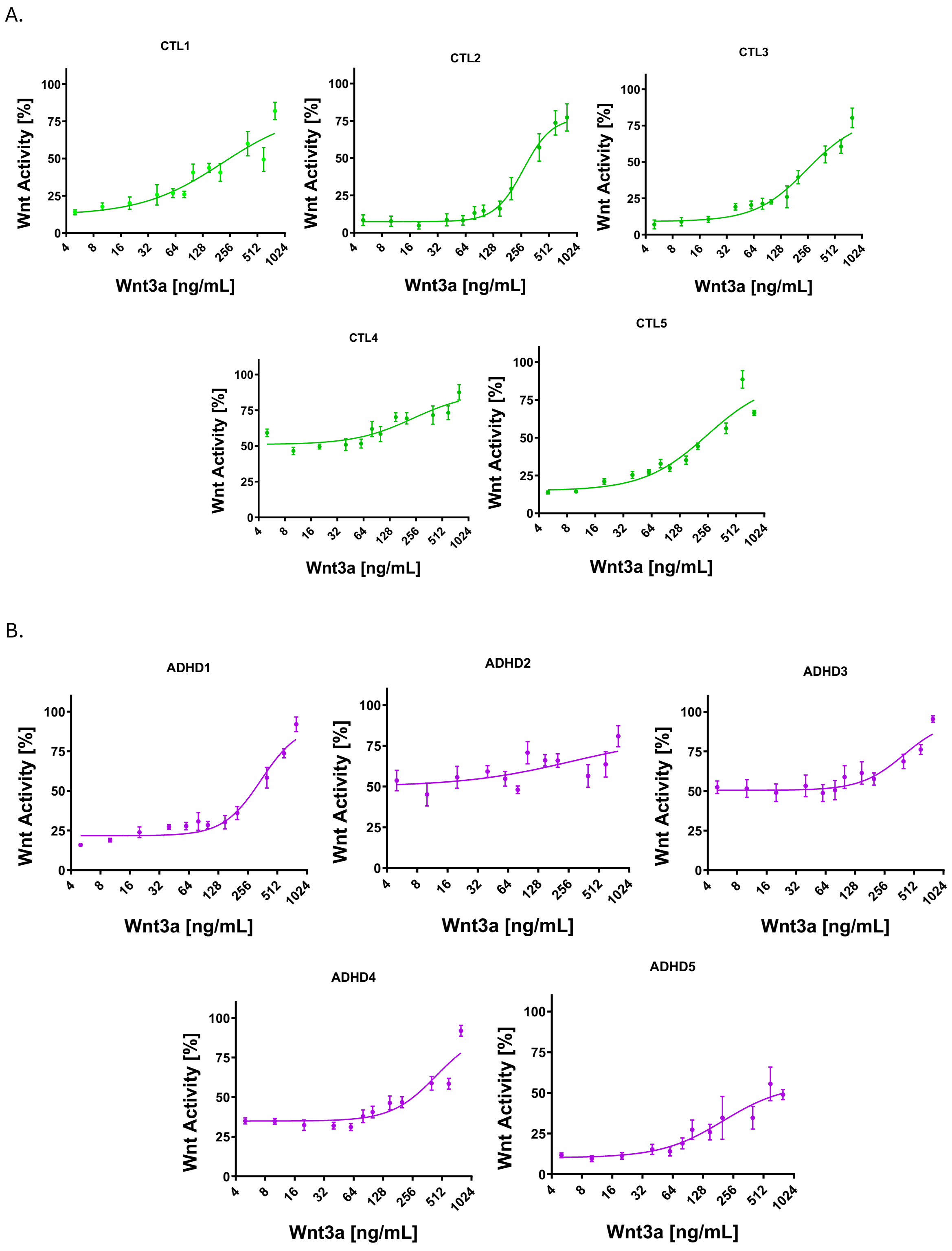

Supplementary Figure 3. Individual dose-response curves after treatments with increasing concentrations of Wnt3a in control (A) and ADHD (B) cell lines. The curves represent mean ± SEM considering triplicates of two independent experiments per NSC line.

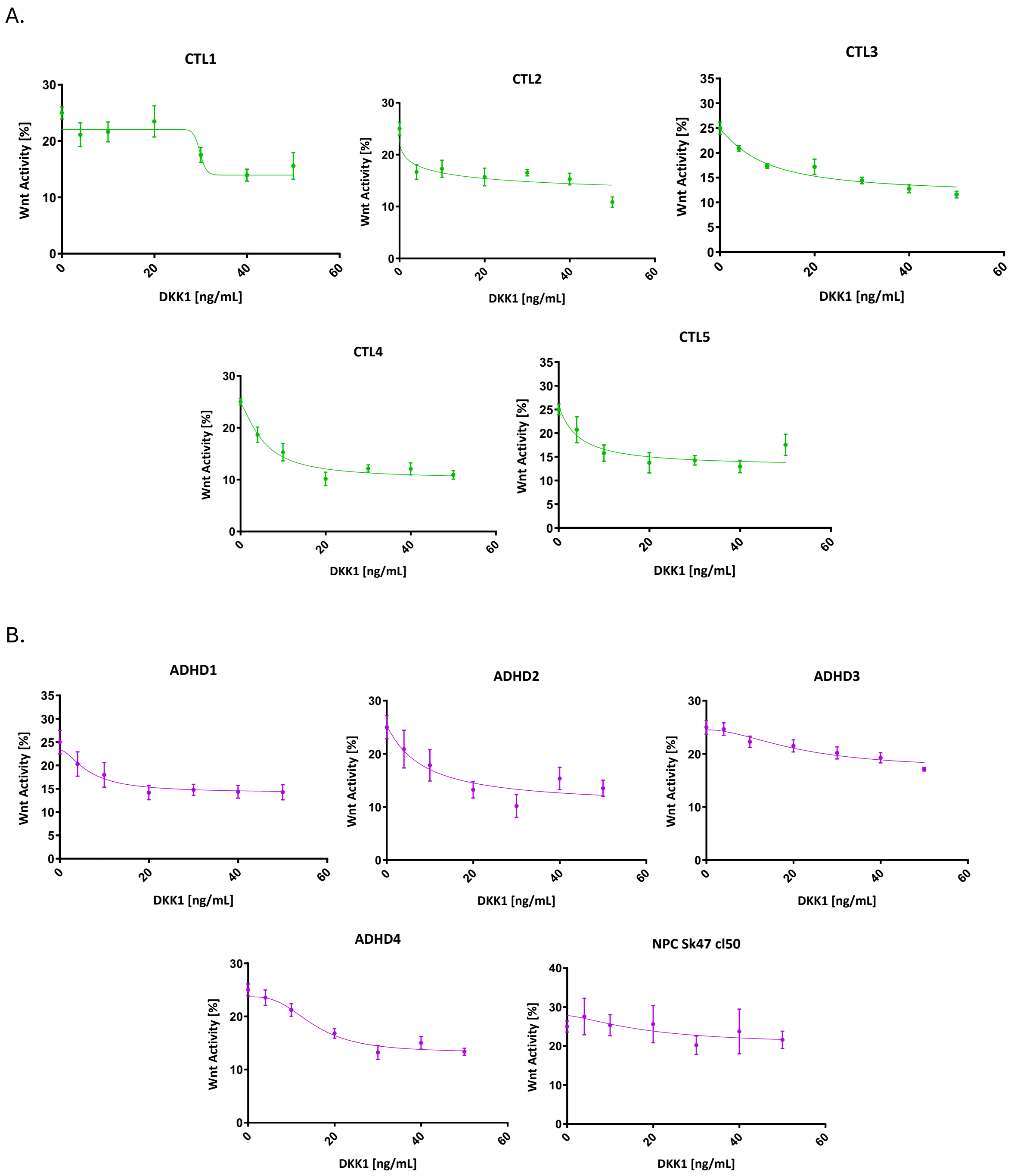

Supplementary Figure 4. Individual dose-response curves after treatments with increasing concentrations of DKK1 in control (A) and ADHD (B) cell lines. The curves represent mean ± SEM considering triplicates of two independent experiments per NSC line.

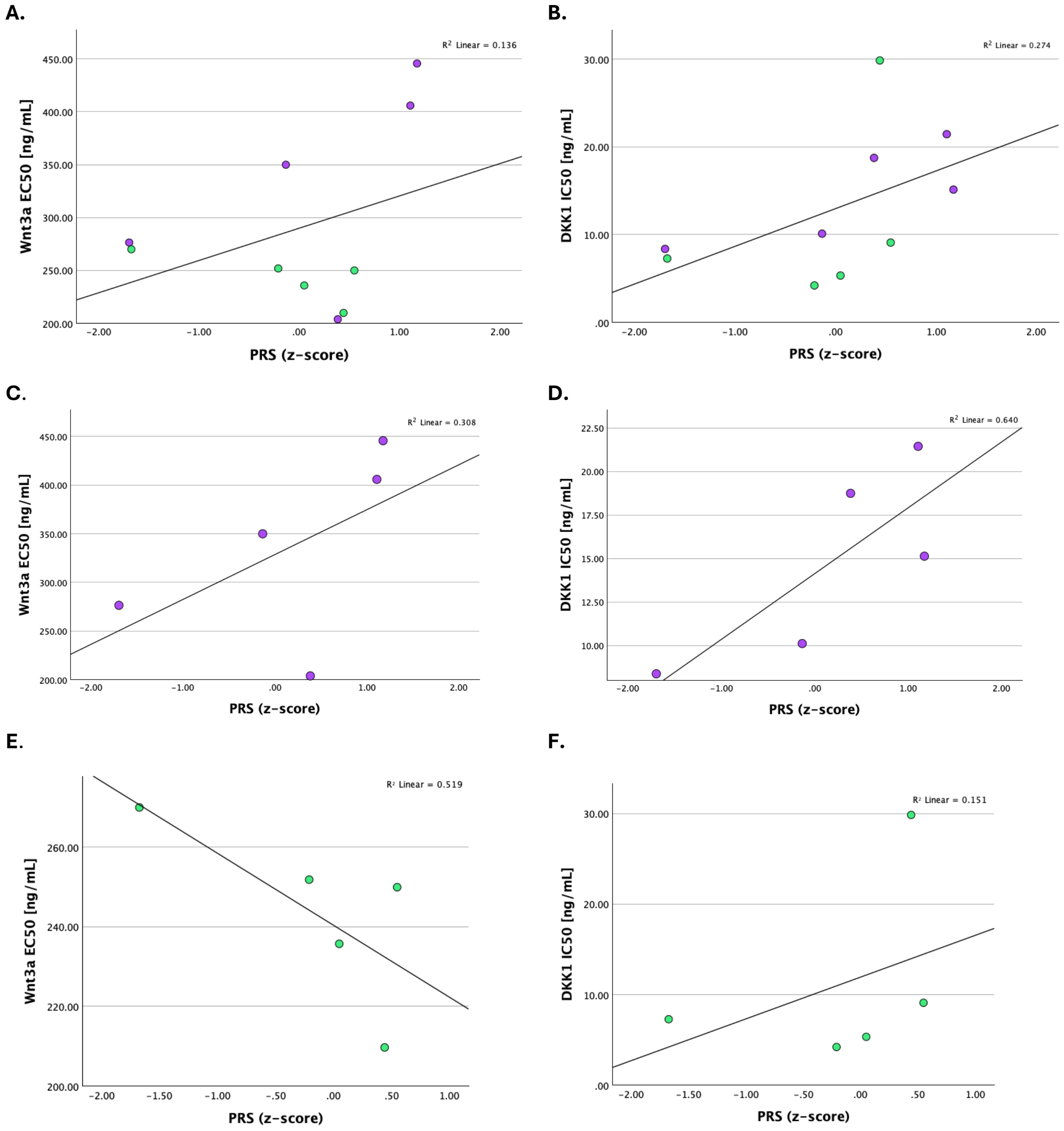

Supplementary Figure 5. Correlation analyses between Wnt-related parameters and genetic predisposition to ADHD. A) No correlation was observed between ADHD-PRS and EC_{50} values of Wnt3a was seen. Spearman's ρ = 0.139; *p* = 0.701. B) A significant positive correlation was observed between ADHD-PRS and DKK1 IC_{50} values (Spearman's ρ = 0.648; **p* = 0.043). When adult ADHD cell lines were analyzed excluding the controls, no correlation was observed between ADHD-PRS and EC_{50} (C) or IC_{50} (Spearman's ρ = 0.700; *p* = 0.188 for both EC_{50} and IC_{50}) (D). The same pattern of response was observed for the control group (Spearman's ρ = -0.700; *p* = 0.188 for EC_{50} and Spearman's ρ = 0.600; *p* = 0.285 for IC_{50}) (E-F). Purple dots represent aADHD while the green dots represent aControl.

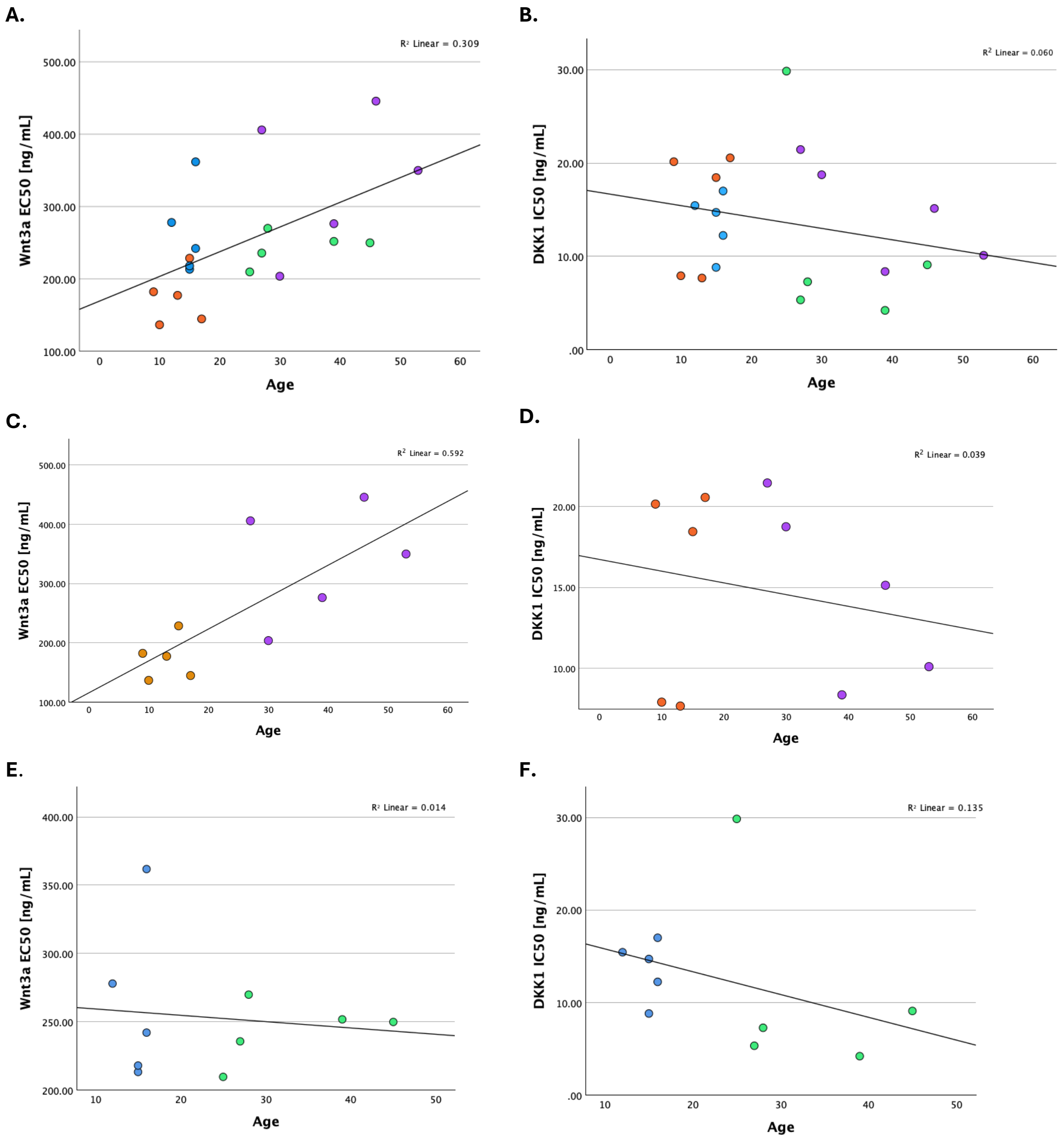

Supplementary Figure 6. Correlation analyses between Wnt-related parameters in NSCs and age of the subjects from the present study and from Walter *et al*. 2024. A) Significant positive correlation between Wnt3a EC$_{50}$ values and age. Spearman's ρ = 0.556; *p = 0.011. B) An opposite, although non-significant, tendency was observed for the correlation between DKK1 IC$_{50}$ values and age. Spearman's ρ = 0.164; p = 0.469. When pediatric and adult ADHD cell lines were analyzed without the controls, a significantly positive correlation between age and Wnt3a EC$_{50}$ values was seen (Spearman's ρ = 0.745, *p = 0.013) (C), but not between DKK1 IC$_{50}$ (Spearman's ρ = -0.006; p = 0.987) (D). Non-statistically significant and opposing tendencies of correlation were seen for the control cell lines (E, F) in comparison to ADHD (Spearman's ρ = 0.091; p = 0.802 for EC$_{50}$ and Spearman's ρ = -0.518; p = 0.125 for IC$_{50}$). Purple = adult ADHD, green = adult control, orange = ADHD children and adolescents (Walter et al. 2024), blue = children and adolescents from the control group (Walter et al. 2024). Wnt-related findings represent one clone per donor.

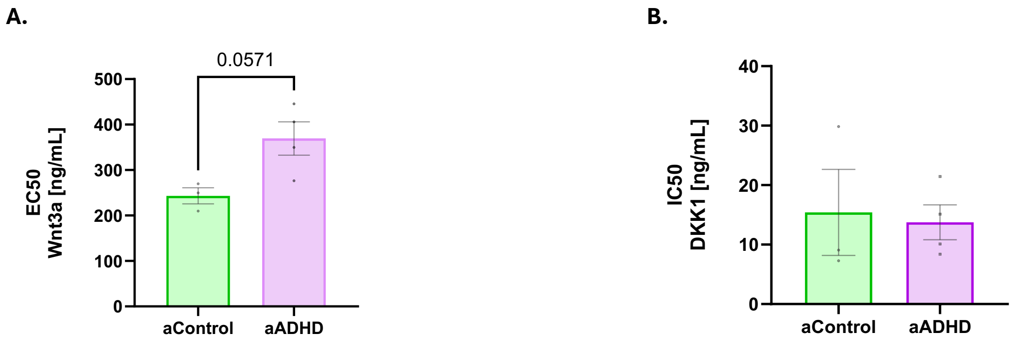

Supplementary Figure 7. Analysis of basal Wnt activity in NSCs derived from only male adult participants. A nominal increase was observed for EC_{50} values of the aADHD group (A) but not for IC_{50} values (B), according to Mann-Whitney tests. *P*-values are displayed in the graphs. Each dot represents the average of two independent experiments from each individual cell line (N = 3 and N = 4 for aControl and aADHD, respectively).
